# Assessment of Heavy Metal Forms and Mobility in Bottom Sediments of Anthropogenically Impacted Freshwater Bodies in Belarus

**DOI:** 10.3390/molecules31081366

**Published:** 2026-04-21

**Authors:** Elizaveta Dorozhko, Witold Kwapinski, Valentin Romanovski

**Affiliations:** 1 Institute of Nature Management, National Academy of Sciences of Belarus, Francisk Skorina, 10, 220076 Minsk, Belarus; 2 Department of Chemical Sciences, Bernal Institute, University of Limerick, V94 C928 Limerick, Ireland; 3 Department of Materials Science and Engineering, University of Virginia, Charlottesville, VA 22904, USA

**Keywords:** bottom sediments, hydrosphere, reservoir, heavy metals, geoaccumulation index, secondary pollution

## Abstract

Bottom sediments in anthropogenically impacted freshwater systems represent a dynamic and poorly constrained source of secondary pollution, where heavy metal mobility, rather than total concentration, controls the release of contaminants into the water column under changing physicochemical conditions. This issue is particularly pronounced in small and medium-sized freshwater systems subjected to sustained anthropogenic pressure, where local hydrochemical conditions and sediment composition strongly influence metal speciation and remobilization dynamics. This study aims to quantitatively assess heavy metal speciation, mobility, and associated ecological risk in bottom sediments of anthropogenically impacted freshwater systems using complementary analytical approaches. The data obtained indicate a pronounced spatial heterogeneity in the total metal content, due to varying degrees of anthropogenic impact on the water bodies. The highest level of pollution was recorded in the bottom sediments of the Chizhovskoye reservoir, where Zn concentrations reach 755 mg/kg, Cr—379 mg/kg, Ni—106 mg/kg, and Cu—158 mg/kg, indicating intense technogenic influence. The bottom sediments of the Loshitsa River are characterized by elevated, but less extreme values: the content of Cu is up to 77 mg/kg, Zn—up to 263 mg/kg, and Mn—up to 418 mg/kg. In contrast to urbanized water bodies, the background site—Lake Sergeevskoye—is characterized by significantly lower concentrations of heavy metals, which confirms its representativeness as a control object. Analysis of the fractional composition showed that Zn and Mn have the largest share of mobile forms, with their concentrations in the mobile phase reaching 12–92 mg/kg and 60–116 mg/kg, respectively, especially under conditions of increased anthropogenic load. A significant portion of Cu and Zn (up to 60–70% of the total content) is associated with organic matter, indicating the important role of the organic matrix in retaining metals and their potential mobilization under changing environmental conditions. Calculation of the geoaccumulation index showed that most of the studied bottom sediments belong to the from uncontaminated to moderately contaminated class, while for Cr and Ni in the Chizhovskoye reservoir, I_geo_ values up to 1.9 are characteristic, corresponding to a moderate level of pollution. The results obtained indicate a significant impact of anthropogenic load on the forms of occurrence and mobility of heavy metals and highlight the role of bottom sediments as an active factor in the secondary pollution of freshwater ecosystems.

## 1. Introduction

Freshwater surface aquatic ecosystems are experiencing an increasing anthropogenic impact associated with urbanization, industrial activity, and the discharge of insufficiently treated wastewater, leading to a deterioration in the quality of water resources. In the Republic of Belarus, this problem is particularly relevant for small rivers and reservoirs operating within settlements and receiving a significant portion of the technogenic load.

One of the key components of aquatic ecosystems that determines their ecological state is the bottom sediments. Under conditions of reduced flow velocity or the presence of stagnant zones, suspended solids settle, forming bottom sediments that accumulate both organic and inorganic polluting components. At the same time, bottom sediments are not a passive geochemical archive but an active environment capable of acting as a source of secondary pollution of the water column when physicochemical conditions change.

Polluted bottom sediments have a complex impact on aquatic ecosystems, including the suppression of self-purification processes, changes in biogeochemical cycles, and a negative impact on benthic communities. The accumulation of pollutants in bottom sediments and their subsequent transfer through trophic chains leads to a decrease in biodiversity and sustainability of aquatic ecosystems [1]. The processes of accumulation and migration of polluting components depend on many factors, including the mineralogical and granulometric composition of sediments, the content of organic matter, the redox potential, and the hydrochemical characteristics of the aquatic environment [2,3,4].

Among the priority pollutants of bottom sediments, heavy metals occupy a special place, belonging to the class of conservative pollutants [5,6]. Heavy metals do not decompose in natural conditions but are able to change their form of existence in the ecosystem under the influence of physicochemical factors such as pH, water mineralization, and temperature [7]. Their ecological hazard is determined not only by the total content but also by the forms in which they exist, since the form of metal binding determines its mobility, toxicity, and bioavailability [2,8].

**Figure 1:**
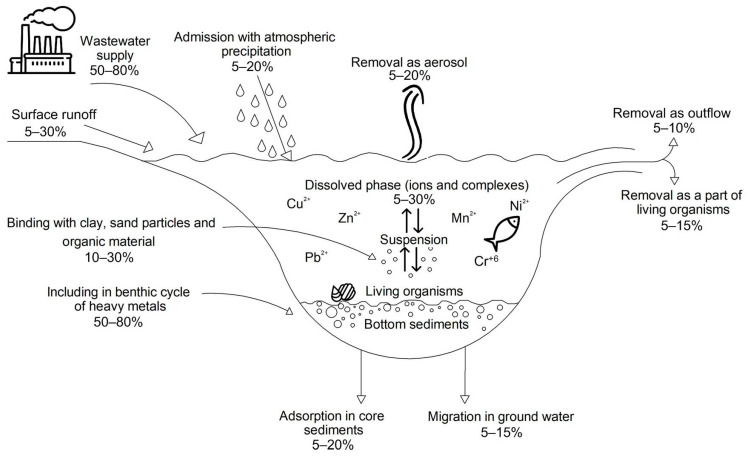
Migration of heavy metals in the aquatic ecosystem.

Anthropogenic input of heavy metals into surface water bodies occurs primarily through discharges of industrial wastewater, atmospheric deposition of solid particles, and runoff from urbanized areas and agricultural lands. As a result, these elements are involved in complex migration processes in the aquatic ecosystem, being redistributed between the water phase, suspended matter, and bottom sediments [6]. In anthropogenically transformed water bodies, bottom sediments become the main reservoir of heavy metals, capable of releasing them back into the water column when environmental conditions change (Figure 1).

The toxic effects of heavy metals on living organisms are well documented and include a wide range of effects—from the inhibition of enzymatic processes to damage to the central nervous system and carcinogenic effects. Historical examples of mass poisonings, such as Minamata disease and Itai-itai disease, have demonstrated the great danger of heavy metal accumulation in aquatic ecosystems and its ability to cause severe socio-ecological consequences [9]. In modern urbanized areas, the risk of chronic exposure to low concentrations of heavy metals remains a relevant environmental problem.

In the surface waters and bottom sediments of Belarus, the most frequently detected heavy metals are Cu, Zn, Pb, Ni, Cr, and Mn, which are characterized by a variety of chemical forms and sources of input (Table S1). For a correct assessment of environmental risk, it is fundamentally important to consider not only the total content of these elements but also their distribution among total, mobile, and organically bound forms, which determine their migratory capacity and potential toxicity.

Despite the extensive body of literature on heavy metal contamination in soils, suspended matter, and large aquatic systems, bottom sediments of small and anthropogenically transformed freshwater bodies remain comparatively underrepresented in studies explicitly addressing metal speciation and mobility as system-defining processes [2,7,8]. Most studies focus on the total content of heavy metals, while the distribution of elements by their forms and their potential mobility are considered fragmentarily or without comparison of different analytical approaches. In addition, the methods used to determine heavy metals are often tested on standardized objects (soils, sewage sludge), which limits the correctness of their interpretation when analyzing bottom sediments, which are characterized by high variability in mineral and organic composition. In the context of anthropogenically burdened small rivers and reservoirs, this leads to uncertainty in assessing the environmental risk and the role of bottom sediments as a source of secondary pollution. Accordingly, the aim of this study is a comprehensive assessment of the forms of occurrence, mobility, and ecological significance of heavy metals (Cu, Zn, Pb, Ni, Cr, Mn) in the bottom sediments of anthropogenic freshwater bodies using and comparing various analytical methods. To achieve this goal, the following tasks were addressed: determining the total and mobile content of heavy metals in bottom sediments; assessing the proportion of metals associated with organic matter; analyzing the mobility of elements and their ability to cause secondary pollution of the aquatic environment; and assessing the degree of contamination of bottom sediments using the geoaccumulation index.

The novelty of this study lies in addressing unresolved questions regarding how sediment-specific properties—particularly redox sensitivity, organic–mineral interactions, and dynamic sediment–water exchange—control heavy metal speciation and mobility in anthropogenically impacted freshwater systems. Unlike soils and sapropels, bottom sediments represent a highly heterogeneous and reactive matrix in which the relationships between total metal content, binding forms, and potential remobilization remain insufficiently constrained. This study therefore provides a targeted assessment of these processes by integrating chemical speciation analysis with mobility assessment and phytotoxicity evaluation to characterize not only metal concentrations but also their functional mobility and environmental significance. In this study, small rivers and reservoirs are considered as water bodies characterized by limited flow capacity, reduced dilution potential, and a high sensitivity to localized anthropogenic inputs. Such systems are particularly prone to the accumulation and remobilization of contaminants, as their sediment–water interactions are more dynamic and less buffered compared to larger river basins and lakes. The study further provides new data for anthropogenically impacted freshwater systems in Belarus, including the Chizhovskoye Reservoir, thereby contributing to the regional understanding of sediment-associated metal behavior and contamination patterns.

The selection of Cu, Zn, Cr, and Ni in this study is based on their contrasting geochemical behavior, binding mechanisms, and mobility in sediment systems. These elements represent a range of interaction pathways with mineral and organic components, allowing for a process-oriented assessment of metal speciation and environmental behavior. The study is therefore focused on elucidating relationships between metal forms and ecological effects rather than providing a comprehensive inventory of all potentially toxic elements.

Despite extensive studies on heavy metals in soils and suspended matter, bottom sediments remain underrepresented as a distinct analytical matrix characterized by high heterogeneity, redox sensitivity, and strong organic–mineral interactions. These features directly affect metal speciation, mobility, and the potential for secondary pollution, yet are rarely addressed in a unified framework. Consequently, conclusions drawn from soil-based assessments cannot be directly transferred to bottom sediments.

The practical significance of the study is related to substantiating the possibility of safely using moderately contaminated bottom sediments as mineral additives and components of soil conditioners and building materials, which allows considering bottom sediments not only as a source of environmental hazard but also as a manageable secondary resource.

## 2. Results and Discussion

### 2.1. Chemical Composition and Speciation of Heavy Metals in Bottom Sediments

The sediment samples represent materials of varying composition and properties. The main characteristics of the collected sediment samples are presented in Table 1. A petroleum odor was present in the bottom sediments of water bodies located within the city limits. The highest moisture content was found in the bottom sediments collected from Lake Sergeevskoye, which suggests that the sediments have a natural accumulation mechanism, as also indicated by the low ash content of the substance (A, %).

**Figure 2:**
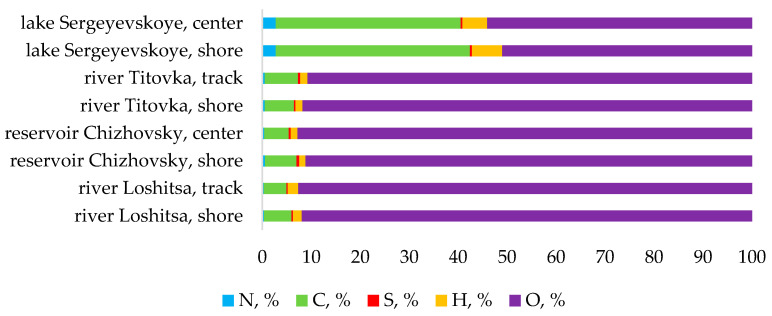
Elemental composition of the bottom sediment samples (wt% of dry matter).

The elemental composition for nitrogen, carbon, hydrogen, and sulfur are presented in Figure 2. The sediments from Lake Sergeevskoye are the richest in organic matter, which indicates the natural character of the formation and accumulation of bottom sediments.

The CHNS/O elemental composition can give us valuable information about how organic matter affects chemical interactions with metals within bottom sediments. The presence of a high quantity of carbon indicates that a large amount of organic matter can form stable metal complexes via chemical functional groups including carboxyl and hydroxyls. Together, these organic–mineral interactions help stabilize the metal elements in a less mobile form, thereby lowering their availability to living organisms and preventing potential damage to plant species grown on that sediment. The difference between the total concentration of metals in the sediment and the observed behavior of biological organisms using the sediment for growth supports this hypothesis; in general, sediments that contain large amounts of organic content will have lower levels of toxicity to living organisms than comparably contaminated sediments containing less organic material will.

**Figure 3:**
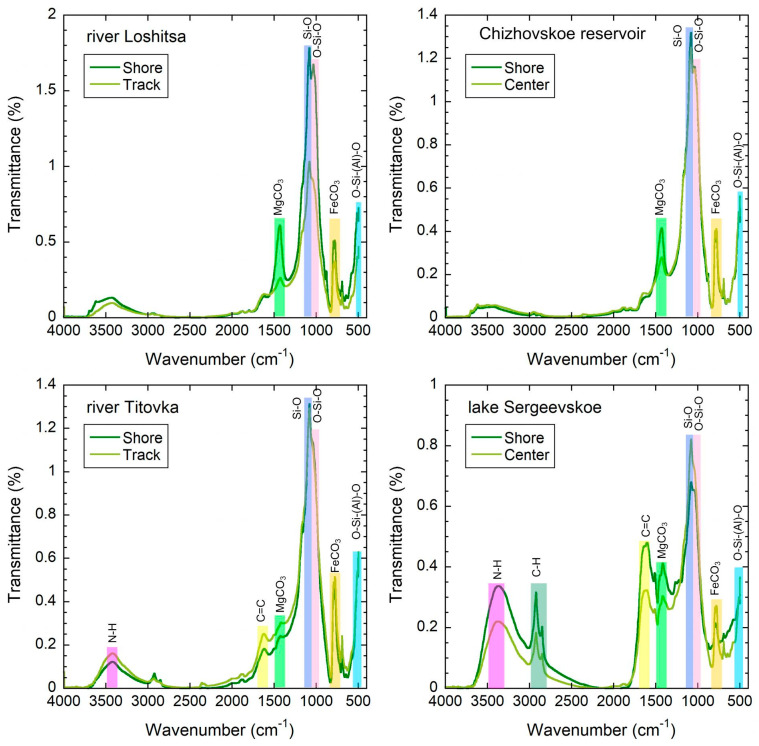
IR-spectra of the bottom sediment samples.

The IR results are presented in Figure 3. IR spectra show the strong presence of organic functional groups found in bottom sedimentary material. Absorption bands in the region of 3200–3500 cm^−1^ indicate hydroxyl group (O–H) stretching vibrations, while the bands observed around 1600–1650 cm^−1^ are due to C=O stretching from carboxyl or conjugated compounds. The potential presence of C–H bending and C–O stretching occurs in the regions of 1400–1500 cm^−1^ and 1000–1100 cm^−1^, respectively, providing evidence for the existence of O-bearing organic compounds; however, it is important to note that these assignments are indicative of the functional groups present and are not definitive assignments for particular molecular structures due to the complexity and heterogeneity of organic matter in the sediment.

The functional groups identified in the IR data support the ability of sediment organic matter to bind to metals via organo-mineral interactions, consistent with the reactivity patterns associated with metal speciation and mobility [10]. This mineral–organic composition of bottom sediments determines their potential role in the processes of secondary pollution of the aquatic environment. The assessment of water quality indicators in this context was considered as a tool for analyzing secondary sources of pollutants entering the water body. Comparing the chemical composition of bottom sediments and water samples allows for predicting the current state of pollution of the water body depending on the ratio of anthropogenic load affecting the bottom sediments and the water phase. The analytical determination of metal content in water samples was carried out using atomic absorption spectroscopy. The content of heavy metals copper, zinc, lead, chromium, nickel, and manganese was experimentally investigated using the atomic absorption method. Five water samples were taken from surface water bodies for analysis. The samples were collected using a submersible bathometer. The results are presented in Table 2.

Based on the data obtained on heavy metal concentrations, it can be concluded that the highest concentrations of heavy metals are found in the Loshchitsa River and the Chizhovskoye reservoir. Cu and Zn exhibit relatively higher concentrations compared to the other analyzed metals, indicating their greater contribution to the overall contamination pattern in the studied sediments. However, it should be noted that both elements are essential micronutrients, and their environmental impact depends on their concentration levels, chemical speciation, and bioavailability rather than total content alone. To quantitatively assess the metal content in bottom sediments, a combined extraction-photometric analytical method was initially used. The results of heavy metal concentration determinations obtained using this method are shown in Table 3. The similarity in concentration trends obtained by the two analytical methods reflects consistency of spatial patterns across total and mobile metal fractions. However, the use of both approaches is essential, as total concentrations alone do not capture differences in metal binding forms and potential mobility, which are critical for interpreting ecological effects observed in the bioassay.

As an alternative analytical approach for determining the heavy metal content, atomic absorption spectroscopy was used. The results obtained using this method are summarized in Table 4.

The main agents responsible for the binding of Cu, Pb, and Zn, both in strongly and weakly bound states, are organic matter and non-silicate minerals. However, their binding to Cu, Pb, and Zn manifests itself differently, depending on the degree of contamination. At different levels of anthropogenic load, organic matter actively participates in the retention of Cu and Pb. In the case of Zn, the dominant role of organic matter is manifested only at high metal concentrations. The measured metal concentrations were evaluated in the context of widely used sediment quality guidelines. For example, Zn concentrations reaching up to 755 mg/kg exceed commonly reported probable effect thresholds (PEC), indicating a high likelihood of adverse ecological effects in the studied sediments. Similar comparisons for other metals suggest that the observed contamination levels in certain sites may be sufficient to induce biological responses, particularly under conditions favoring metal remobilization [11].

The wet ashing method was used to determine the amount of metal bound to organic matter. The results are presented in Table 5.

To characterize the degree of mobilization of trace metals in bottom sediments, a mobility index was used, represented as the percentage of their mobile forms relative to the total content [12]. The results are shown in Table 6. In the presence of organic matter, considered as an independent factor, the mobility of metals, in particular Cu, Zn, Pb, and Mn, increases.

According to [10], the geo-accumulation index is the most comprehensive method for assessing the degree of heavy metal contamination of bottom sediments. This index has seven classes (from 0 to 6) corresponding to different levels of contamination (from uncontaminated to extremely contaminated).

Bottom sediments collected from Lake Sergeyevskoye were used as a background sediment sample due to the natural mechanism of formation and accumulation, as well as due to the minimal anthropogenic impact on the sediments. The calculation results are presented in Table 7.

The correspondence between the I_geo_ value and the class and level of sediment pollution is shown in Table S2. Analysis of the calculated geoaccumulation indices showed that the degree of contamination of bottom sediments in the studied water bodies generally varies from the uncontaminated to moderately contaminated categories, with the severity of contamination depending on the specific element and sampling location. For copper, the index values correspond to a transitional state between no contamination and moderate contamination. In the case of zinc, most samples belong to the uncontaminated category; however, bottom sediments sampled in the coastal zone of the Loshitsa River and the Chizhovskoye reservoir are characterized by a level corresponding to moderate contamination. For lead, all studied samples are also in the range between uncontaminated and moderately contaminated. A more pronounced degree of accumulation was found for chromium: bottom sediments of the Loshitsa River, the coastal and central parts of the Chizhovskoye reservoir, and the Titovka River are classified as moderately contaminated, while the remaining samples belong to the intermediate category. For nickel, a moderate level of contamination was found exclusively in the bottom sediments of the Chizhovskoye reservoir, while other areas are characterized by lower index values. Regarding manganese, all studied bottom sediments are in the range from uncontaminated to moderately contaminated, with a predominance of characteristics corresponding to the background state. While the geoaccumulation index (I_geo_) has been useful in comparing different areas of contamination, it should be noted that the index was originally designed specifically for soils, and therefore, it does not necessarily account for all the dynamic processes occurring in bottom sediments. Focusing on specific factors such as sediment re-suspension and seasonal variations in redox potential can greatly affect the distribution of contaminant metals, as demonstrated by the exploration of multiple potential methods of analysis on a composite sample taken from the same location. In order to accurately assess contaminant distribution, it is essential to use I_geo_ in conjunction with other indicators, such as the results of metal speciation analysis. In the context of this study, the I_geo_ has been used as a conventional method for contextually assessing contaminated sites. However, it is important to note that an index based solely on total concentrations of metals does not address issues like the identity of metal species and how each species can exist in various forms, nor does it account for the mobility of metals, both of which are necessary when evaluating the overall impact of contamination on ecological and environmental health. Because of this limitation, the present study will not include several methods for assessing contaminated sediments based upon I_geo_, but rather, will utilize Igeo along with speciation analyses and biological assessment in order to provide a more robust and dynamic evaluation of sediment contamination.

Within the Chizhovskoye reservoir, there was significant spatial variation in observed metal concentrations between shoreline (close to the land) locations and central (in the water column) locations. This variability is attributed primarily to the deposition and sedimentation processes and the difference in hydrodynamic conditions between these two locations. For example, shoreline sediments tend to accumulate more metals due to their location being directly adjacent to local anthropogenic (human) inputs (i.e., urban runoff and wastewater), which generally results in much higher concentrations of metals (Zn, Cr, Cu) than in central sediment deposits. Central sediment deposits are composed of fine particles that serve as a sink for metals that have been redistributed from their sources through sedimentation.

The difference in metal concentrations measured between riverbank (near land) versus riverbed (in water) samples from all of the sampling sites (Table 3 and Table 4) were found to vary two to three times from each other, rather than being inconsistent samples (i.e., laboratories or protocols used to run samples); it is common in freshwater systems impacted by anthropogenic activities (i.e., adding contaminants), where the loading from outside sources and the transport of sediment result in a heterogeneous pattern of metals found within one waterbody.

The values of total heavy metal content in bottom sediments of anthropogenically transformed water bodies in Belarus obtained in this study are consistent with pollution levels previously recorded for other urbanized and industrially modified freshwater ecosystems. In particular, the maximum concentrations of zinc (up to 755 mg/kg) and chromium (up to 379 mg/kg) found in the bottom sediments of the Chizhovskoye reservoir are within the range of values characteristic of the bottom sediments of the Warta River (Poland), where in areas of intense anthropogenic impact, the Zn content reaches 600–800 mg/kg, and Cr—300–400 mg/kg [6]. This comparison indicates a comparable level of technogenic load and confirms the representativeness of the results obtained in a broader regional and international context. Similar levels of heavy metal contamination in bottom sediments have been observed in certain sections of the Nile River, where the concentrations of Cu, Zn, and Cr significantly increase in areas with urban and industrial pollution [2]. This indicates that the levels of sediment contamination identified in the studied water bodies of Belarus are consistent with global trends for anthropogenically transformed freshwater ecosystems.

The distribution of heavy metals across different forms of occurrence is of particular importance for assessing ecological risk. This study found that a significant proportion of Cu and Zn (up to 60–70% of the total content) is associated with organic matter in bottom sediments, especially under conditions of high anthropogenic load. Similar behavior of metals has previously been observed in the bottom sediments of large rivers and reservoirs, where organic matter acts as an effective accumulator of metals, capable of both stabilizing them in the sediments and promoting secondary mobilization when redox conditions change [2,8]. In urbanized water bodies, the accumulation of organically bound forms of Cu and Zn can be considered an indicator of technogenic impact and potential ecological instability of bottom sediments.

The revealed high values of mobile forms of Zn and Mn (up to 90 and 116 mg/kg, respectively) indicate that the bottom sediments of the studied water bodies are not geochemically inert, but are capable of acting as an active source of secondary pollution of the water column. It has previously been shown that fluctuations in pH, mineralization, and redox potential can lead to intensive redistribution of metals between the solid and aqueous phases, which leads to a deterioration in water quality even in the absence of current sources of pollution [1,7]. The results obtained confirm that for small rivers and reservoirs with an unstable hydrological regime, the contribution of bottom sediments to the formation of the chemical composition of water can be comparable to the direct anthropogenic input of pollutants.

It should be noted that metal mobility does not necessarily imply immediate bioavailability but rather reflects the potential for remobilization under changing environmental conditions. Variations in redox potential, pH, and organic matter decomposition may trigger the release of previously immobilized metal fractions. Therefore, mobile forms identified in bottom sediments represent a latent risk of secondary contamination rather than direct ecological exposure. This distinction is critical for interpreting sediment quality assessments. While cadmium is recognized as a highly toxic element, its exclusion from the present study does not affect the interpretation of the identified relationships between metal speciation, mobility, and phytotoxicity, which are governed by general sediment–metal interaction mechanisms.

### 2.2. Phytotoxicity

**Figure 4:**
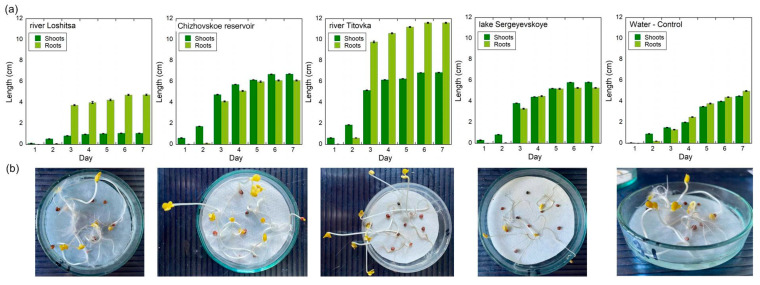
Length of roots and shoots (**a**), photographs of seedlings on day 7 (**b**).

The results of phytotoxicity tests of bottom sediments are presented in Figure 4. In general, most of the studied samples did not show a pronounced phytotoxic effect and were characterized by germination and root growth rates comparable to the control variant. An average degree of phytotoxicity (hazard class 3) was recorded exclusively for bottom sediments of the coastal zone of the Loshchitsa River, which indicates the presence of factors capable of having a suppressive effect on the early stages of plant development [13].

Analysis of morphometric indicators showed that the greatest root length was observed in plants grown on bottom sediments of the coastal zone of the Chizhovskoye reservoir (6.1 cm) and from the channel of the Titovka River (11.62 cm). The minimum values of root length were recorded for sediments of the coastal zone of the Loshchitsa River (4.75 cm) and the central part of Lake Sergeyevskoye (4.16 cm), which indicates less favorable conditions for plant growth in these substrates.

**Figure 5:**
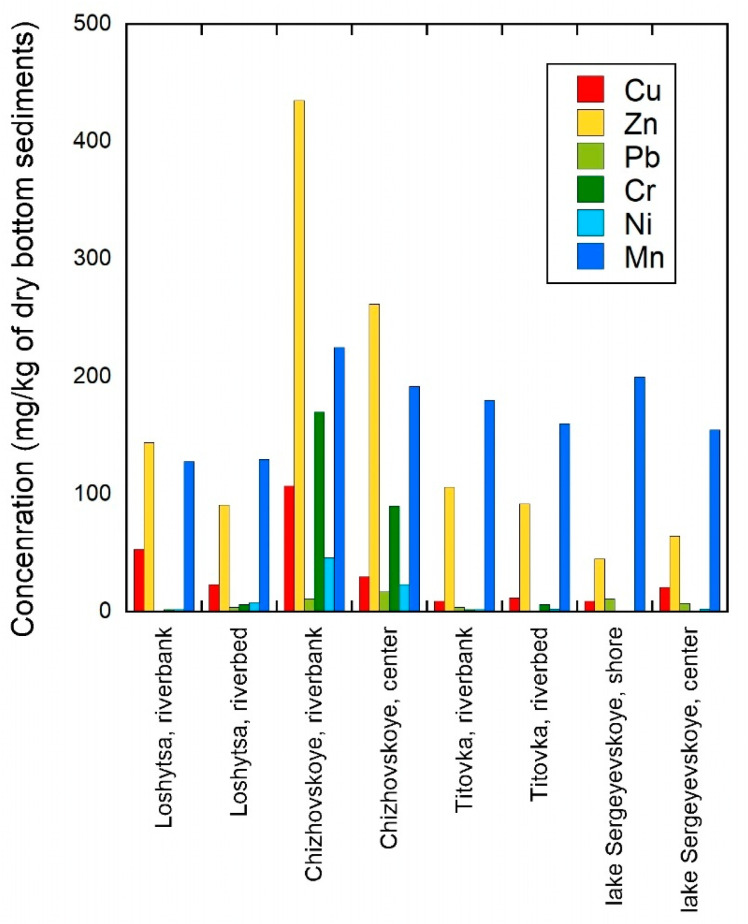
Results of metal content in plant material.

A comparative analysis of phytotoxicity indicators with the results of determining the heavy metal content in plant material (Figure 5) showed that pronounced suppression of root system growth is not observed in all cases of maximum metal accumulation in plant tissues. For example, despite the highest levels of accumulation of Cu, Zn, Cr, and Ni in plant material grown on bottom sediments of the coastal zone of the Chizhovskoye reservoir, no phytotoxic effect was observed in this case. An apparent discrepancy between the accumulation of metals in plant tissues and plant injury means that there is no simple relationship between these two things. Although the tissue concentrations of Cu, Zn, Cr, and Ni in certain areas are much higher than normal, there is no growth inhibition associated with these metals because they are associated with relatively stable organo-mineral fractions that do not release the biologically active forms of these metals necessary to cause a toxic response. In addition, some of the complexes that form with metals in the soil may be partially dissolved and, as a result, help in the uptake of metals through rhizosphere processes (e.g., root exudates) without causing any injury or toxicity to the plant. This distinction means that although metal accumulation relates to total uptake, phytotoxicity is based on the presence of the reactive and bioactive forms of the same metals.

In contrast, the bottom sediments of the Loshchitsa River were characterized by both increased accumulation of Cu, Zn, and Mn in plant material and reduced root length, indicating a higher proportion of bioavailable forms of metals and their potential phytotoxicity. Similarly, the reduction in plant growth on the bottom sediments of the central part of Lake Sergeevskoye may be associated not so much with the absolute concentrations of metals, but rather with the characteristics of the organic matrix of the bottom sediments and the possible mobilization of individual elements—in particular Mn—when the conditions of the root environment change. It should be noted that at certain concentrations, trace elements, including Cu, Zn, and Mn, are able to perform a physiological function and participate in the regulation of plant metabolic processes, acting as cofactors in enzymatic reactions. In some cases, this can lead to a stimulating effect on plant growth, which has been previously shown in published studies, including for systems with elevated metal content but limited bioavailability.

Thus, the results of phytotoxicity tests confirm that the phytotoxic effect of bottom sediments is determined not by the total content of heavy metals, but by their binding forms and bioavailability. Bottom sediments with a high content of metals bound in stable organic and mineral complexes may not exhibit toxic effects on plants, while in the presence of more labile forms, even moderate concentrations of metals can lead to growth inhibition. The obtained data emphasize the need to consider the forms of metal occurrence when assessing the ecological hazard of bottom sediments.

The patterns of bioaccumulation and distribution of heavy metals identified in this study mirror those previously demonstrated for sediment-plant systems. Furthermore, the fact that Zn and Cu were the most abundant metals in the present study correlates with the metals’ distribution and bioavailability in anthropogenic-impacted locations, where Zn is generally the most bio-available followed by Cu and Cr [14]. This relationship is largely due to the higher mobility of Zn in sediment and their being less tightly bound to stable mineral phases than Zn, which is reflected in its higher bioavailability.

Similarly, although the accumulation of metals into plant tissue resulted in no discernable phytotoxicity, this is not uncommon. As identified in previous studies of sediment–plant systems, metals can be partially accumulated but remain within compartments (e.g., cell walls or vacuoles) that do not have a high physiological activity and therefore exhibit limited toxicity [15]. For example, Cu and Zn can be sequestered or immobilized within root cell structures, which would limit the transport of metals to metabolically active plant tissues, thereby producing little to no phytotoxicity.

In addition, the concentrations obtained in this study are similar to those that have been found in polluted wetlands, where Cu and Zn concentrations approximately ranged from 30 to 70 mg/kg and showed significant accumulation when compared to background levels (up to ~75% increase) [16]. This supports the interpretation of the measured system as having been affected by anthropogenic inputs, acting as a sink for trace metals.

Moreover, moderate levels of phytotoxicity in this study are consistent with findings from previous studies that indicate that essential elements such as Cu and Zn may accumulate to relatively high concentrations while not exhibiting a high level of toxicity because of the plant’s tolerance mechanisms and homeostatic regulation [17]. This further indicates that total metal concentrations alone cannot predict the biological impacts without taking into consideration factors such as metal speciation and metal bioavailability.

Overall, the findings of this study support the current understanding that: (i) bottom sediments are both sinks and secondary sources of heavy metals, (ii) Zn and Cu are commonly dominant in anthropogenically altered systems, and (iii) accumulation of metals in plants does not necessarily indicate high levels of bioavailability or toxicity, particularly when there is strong organo-mineral binding.

The current study presents a spatial assessment of redox dynamics, metal mobility and sediment–water interactions under very specific environmental parameters and does not take into consideration potential seasonal variability; this may have an impact on redox dynamics, metal mobility, and sediment–water interactions. The results of the phytotoxicity tests provide an understanding of what biological response can be expected. However, the study does not explore the potential for trophic transfer to occur in aquatic food webs. Despite these limitations, the results from this study may serve as a reliable basis for evaluating the potential for metals to be mobilized and bioavailable from bottom sediments. Bottom sediments are also critical variables in assessing ecological risks and their potential transfer to biota as the environment changes.

## 3. Material and Methods

### 3.1. Characteristics of the Objects Under Study

**Figure 6:**
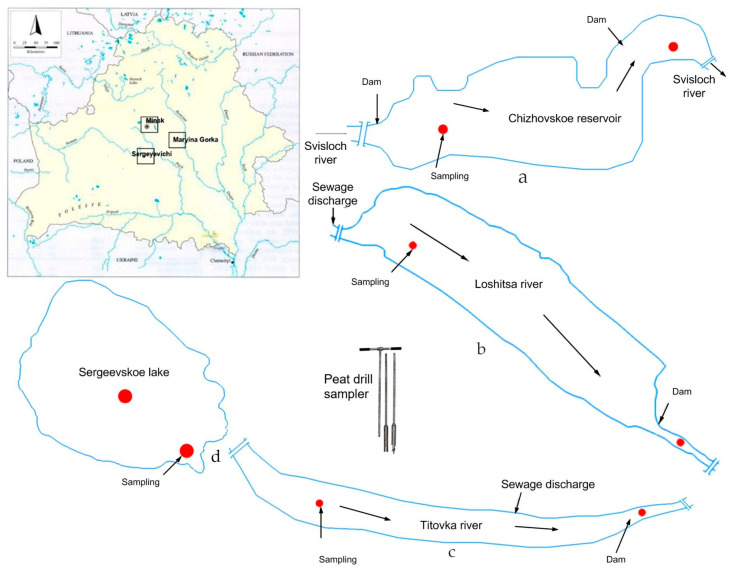
Water objects of sampling of the bottom sediments: a—Chizhovsky reservoir; b—river Loshitsa; c—river Titovka; d—lake Sergeyevskoye.

The water bodies selected for bottom sediment sampling were located within the city of Minsk and the Minsk region, including the Loshitsa River (Minsk), the Chizhovskoye Reservoir (Minsk), the Svisloch River (Minsk, near the zoo), the Titovka River (Maryina Gorka, Pukhovichi district, Minsk region), and Lake Sergeevskoye (Sergeevichi village, Pukhovichi district, Minsk region) (Figure 6).

The Loshitsa River is a right-bank tributary of the Svisloch River and flows into it approximately 1 km upstream from the Chizhovskoye Reservoir. Under natural hydrological conditions, the length of the waterway was about 12 km with a catchment area of 67 km^2^. As a result of the operation of an underground water intake in the Petrovshchina area, the length of the river has been significantly reduced and currently does not exceed 7 km. In the area of Semashko Street, upstream from the confluence with the Myshka River, the Loshitsa receives runoff from the Slepyanka storm sewer system, which accumulates surface and treated wastewater from enterprises in the western part of the city. This anthropogenic inflow largely compensates for the loss of natural water flow in the middle and lower reaches of the river.

The Chizhovskoye Reservoir is located in the southeastern part of Minsk and was built in 1951 on the Svisloch River because of technical water supply needs. It experiences significant anthropogenic impact, has been in long-term operation, and operates under difficult conditions, passing river water from the Svisloch River and all wastewater from the city.

The water network of Minsk is formed by a system of regulated watercourses, with the Svisloch River at its center. This water body is part of the Vileika–Minsk water management complex, commissioned in 1976 for the redistribution of water resources to serve urban water supply, the industrial sector, irrigation needs, and integrated runoff management. The intensive involvement of the Svisloch River in economic activity has led to a consistently high level of anthropogenic load within its basin. Currently, 40 water-using enterprises operate in the river’s catchment area, discharging wastewater directly into the basin’s water bodies, which significantly transforms the river’s hydrochemical regime.

The Titovka River is a secondary watercourse, formed by an artificial hydraulic connection with the Ptich River via a drainage canal laid along the left-bank floodplain. The source of the Titovka is located approximately 1.5 km southeast of the village of Rusakovichi. The river flows into the Svisloch River from the right bank at the 109 km mark from the mouth, in the area adjacent to the eastern part of the agro-town of Pukhovichi. The length of the river is 33.0 km, and its catchment area is 372 km^2^. For 16.0 km from its source near the village of Zagay, the river is canalized. Within the city of Maryina Gorka, a group of artificial reservoirs has been created on the Titovka River.

The local hydrological reserve “Sergeevichsky” is located in the northwestern part of the Pukhovichi district of the Minsk region. In its center is Lake Sergeevskoye, and the total area of the reserve is 2006 hectares. This reserve was created to stabilize the water regime of Lake Sergeevskoye and for the ecological rehabilitation of the worked-out areas of the Rady-Golyshevka peat deposit.

Sampling was conducted during the spring period of 2025, under stable hydrological conditions to ensure comparability between sites. Bottom sediment samples were collected from the upper layer (0–10 cm) using manual grab sampling with non-metallic tools to avoid contamination. At each site, samples were taken from representative zones (shoreline, riverbed, or central depositional areas) depending on local hydromorphological conditions. The sampling design was intended to capture spatial variability in sediment composition under different levels of anthropogenic influence. The collected samples were homogenized, air-dried, and further prepared for physicochemical analysis. Water samples were collected from the same locations as sediment samples using a submersible bathometer. The samples were stored in clean polyethylene containers and analyzed for dissolved metal concentrations using atomic absorption spectroscopy.

### 3.2. Methods for Analyzing Samples

The determination of heavy metal concentrations in the studied samples was performed using two analytical approaches: a combined method based on photometry and titrimetry, and atomic absorption spectroscopy [18,19,20].

To determine the total metal content, the samples were subjected to decomposition using strong acids (HNO_3_/HCl, Ultra-pure, for ICP-MS, Thermo Fisher Scientific, Waltham, MA, USA). Extraction of mobile metal forms was performed using ammonium acetate (Ultra-pure, Sigma-Aldrich, St. Louis, MO, USA) buffer (pH 4.8) according to established procedures for assessing weakly bound and exchangeable metal fractions in soils and sediments [21]. This approach provides an operational estimate of the mobile fraction, including metals associated with ion-exchange sites and weak surface complexes. It should be noted that this method does not strictly correspond to a specific sequential extraction scheme (e.g., Tessier or BCR), but represents a simplified procedure for evaluating environmentally relevant mobile metal forms.

The main technical requirements for the material include the moisture content and ash content of the sample. The essence of the method for determining the moisture content of bottom sediments was to determine the loss of moisture during drying at a temperature of 105 ± 2 °C to a constant mass. Grain-size distribution was not determined in this study. However, sediment heterogeneity was considered qualitatively based on field observations (e.g., silty, gravelly, organic-rich textures), which were considered during data interpretation.

The ash content of the samples was determined using the same crucibles that were used in the moisture measurement stage. After drying, the sediment samples were calcined in a muffle furnace at a temperature of 500–600 °C for 1 h. After the heat treatment, the crucibles were cooled in a desiccator to room temperature, after which they were weighed on an analytical balance.

A VARIO EL CHNS analyzer (ELEMENTAR, Langenselbold, Germany) was used to determine the elemental composition of the bottom sediments.

To clarify the composition of the mineral and organic parts of the substances in the bottom sediments, the method of IR spectroscopy was used on a IRPrestige-2 Fourier transform infrared spectrometer (Shimadzu, Kyoto, Japan). The method for preparing bottom sediment samples for analysis involved grinding 1 mg of the substance under study in an agate mortar with 300 mg of dry KBr.

Two methods of physicochemical analysis were used to determine the heavy metal content. The first method was sequential, consisting of the extraction and photometric determination of each metal.

Bottom sediment samples were dried to an air-dry state at a temperature not exceeding 40 °C. The dried samples were spread on a film, large lumps were broken up, and inclusions were removed. Then they were ground in a porcelain mortar and sieved through a sieve with round holes 1–2 mm in diameter.

**Figure 7:**
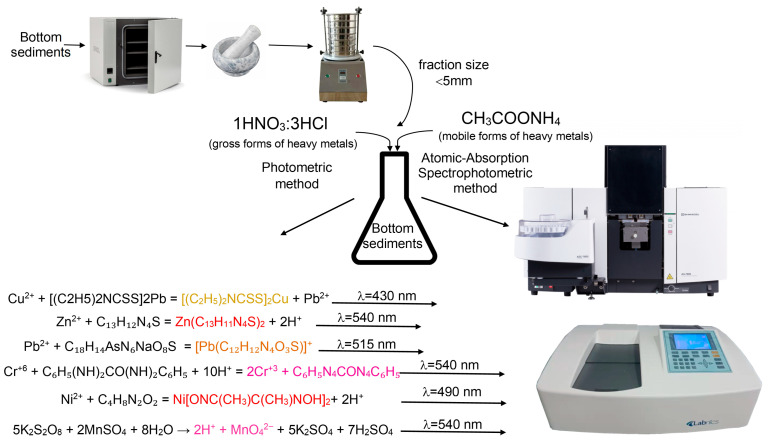
Scheme of methods for studying the concentration of heavy metals in bottom sediments.

The general scheme for determining heavy metals in bottom sediments is shown in Figure 7.

Bottom sediment samples weighing (5.0 ± 0.1) g were placed in beakers, and 50 mL of extracting solution was added to each: for total extraction, a HNO_3_:HCl solution; and for mobile forms, CH_3_COONH_4_ with pH = 4.8.

The extraction–photometric method was used to determine Cu (II) ions in the bottom sediment extract, based on the interaction of the copper solution with lead diethyldithiocarbamate in a CCl_4_ medium, resulting in the formation of yellow-brown copper diethyldithiocarbamate, soluble in the organic solvent layer. Light absorption was measured at λ = 430 nm. The copper content was determined from the average value of optical density (D) of the sample and the calibration curve. The copper content in the sample was calculated using Equation (1):

(1) $$C=\frac{m_{K}\cdot V}{m\cdot V_{a}},$$

where C is the concentration of copper in the bottom sediment sample, mg/kg; m_K_ is the mass of copper determined from the calibration curve, μg; V is the volume of the bottom sediment extract, in mL; and V_a_ is the volume of the aliquot taken for analysis, mL.

The determination of Zn (II) content was carried out by the dithizone method (photometric method). The method is based on the formation of a red-colored zinc compound with dithizone, followed by the extraction of zinc dithizonate into a CCl_4_ layer (at pH = 4.5–4.8) at λ = 540 nm. The concentration of other metals was calculated identically to that of copper using Formula (1).

The determination of Pb (II) content was carried out by the plumbon method (photometric method). The method is based on the formation (at pH = 7.0–7.3) of a lead compound with sulfarsazen, colored yellow-orange. Lead is pre-extracted with dithizone in CCl_4_ (at pH = 9.2–9.6). The resulting lead dithizonate is destroyed with hydrochloric acid. In this case, lead ions pass into an aqueous solution, in which lead is determined. The optical density of the complex compound solution was measured at λ = 515 nm.

The method for determining Cr (VI) is based on measuring the light absorption at λ = 540 nm of the colored (red-violet) complex compound of 1,5-diphenylcarbazonate Cr (III), formed as a result of the redox reaction of Cr (VI) with 1,5-diphenylcarbazide in an acidic medium, and determining Cr (VI) from the optical density value of the solution. The photometric method for determining the concentration of Ni(II) ions is based on the interaction of Ni (II) ions in a weakly ammoniacal medium, in the presence of a strong oxidizing agent, with dimethylglyoxime to form a pink complex compound. The maximum light absorption corresponds to a wavelength of λ = 490 nm.

The photometric determination of Mn(II) involves the catalytic oxidation of manganese compounds with potassium persulfate or sodium persulfate to permanganate ions, followed by measuring the optical density of the solution and calculating the mass concentration of manganese in the sample at a wavelength of λ = 540 nm.

Another method for determining heavy metals was atomic absorption spectroscopy. Samples were analyzed using a Shimadzu Atomic-Absorption Spectrophotometer AA-7800.

To determine the amount of metal bound to organic matter, the wet ashing method was used. A sample of bottom sediment was subjected to ashing in a Thermo Scientific CP1065058 Programmable Muffle Furnace at 700 °C, followed by dissolution of the ash in concentrated HNO_3_. The resulting suspension was quantitatively transferred to 25 mL volumetric flasks, and the amount of metals released from organometallic complexes was then determined. It should be noted that this procedure does not selectively isolate strictly organic-bound metals, but rather provides an operationally defined fraction reflecting the contribution of organic matter and thermally labile components to metal binding. Possible alterations of mineral phases at elevated temperatures were considered in the interpretation of results.

The combination of atomic absorption spectroscopy (AAS) and extraction–photometric analysis was employed to differentiate between total metal concentrations and operationally defined mobile forms. While AAS provides quantitative determination of total metal content, the extraction–photometric approach targets the fraction of metals associated with more labile and potentially bioavailable forms. This distinction is critical for assessing metal mobility and environmental impact.

The mobility of metals in bottom sediments was assessed as the percentage of mobile forms relative to total metal content, Equation (2).

(2) $$K_{M}=\frac{C_{M}}{C_{V}}\cdot 100,$$

where K_M_ is the mobility coefficient of the forms, %; C_V_ is the gross metal content, mg/kg; and C_M_ is the mobile content, mg/kg.

The mobility coefficient (K_M_) quantifies how much metal exists in the mobile fraction compared to the total concentration of metals in bottom sediments. The mobile fraction (C_M_) represents the metals released in an ammonium acetate buffer (pH 4.8) and includes mostly weakly bound/exchanged, ion-exchange-site-associated metals as well as metals associated with labile surface complexes. Thus, K_M_ provides a way to estimate the potential for metals to move in the environment and their relative availability under different physicochemical conditions. Higher values of K_M_ indicate that there are a large percentage of metals in easily mobilized forms, which may lead to secondary pollution and an increased risk to the environment.

The assessment of secondary pollution of the water body was carried out using the distribution coefficient of trace metals “bottom sediments-water”, calculated as the ratio of the element’s content in bottom sediments to its content in water using Equation (3).

(3) $$K_{R}=\frac{C_{DO}}{C_{W}},$$

where K_R_ is the distribution coefficient; C_DO_ is the total metal content in the bottom sediment, mg/L; and C_W_ is the total metal content in water, mg/L.

The assessment of metal migration from bottom sediments to plant material was carried out using the “bottom sediment-plant” distribution coefficient, which was calculated as the ratio of the metal content in the bottom sediment and in the grown plant material using Equation (4).

(4) $$K_{P}=\frac{C_{DO}}{C_{R}},$$

where K_P_ is the distribution coefficient; C_DO_ is the total metal content in the bottom sediments, mg/kg; and C_R_ is the total metal content in plants, mg/kg. For reference, the conventional bioaccumulation factor (BAF) corresponds to the inverse of this ratio (C_R_/C_DO_).

The anthropogenic load on the bottom sediments with respect to metals was calculated using the geoaccumulation index (I_geo_). The geo-accumulation index is calculated using Equation (5):

(5) $$I_{\mathrm{geo}}=\frac{\log_{2}\left(C_{n}\right)}{1.5\left(B_{n}\right)}$$

where I_geo_ is the geoaccumulation index; C_n_ is the measured concentration of the heavy metal; B_n_ is the background content of the heavy metal [22]; and 1.5 is a coefficient that minimizes the effect of possible background variation.

### 3.3. Phytotoxicity Assessment

Phytotoxicity of bottom sediments was evaluated using a seed germination bioassay with *Raphanus sativus* as a test species. For each sample, 20 viable seeds were placed in Petri dishes on filter paper. Distilled water was used as a control, while aqueous extracts of bottom sediments were applied to experimental samples (5 mL per dish).

The dishes were incubated at 20–23 °C under controlled conditions. Germination rate was assessed after 3 days, and seedling growth was monitored over a 7-day period. Root length was measured as the primary morphometric indicator of phytotoxicity.

### 3.4. Quality Assurance and Quality Control (QA/QC)

All analytical measurements were performed in triplicate (n = 3), and results are reported as mean values. Method precision was evaluated using relative standard deviation (RSD) derived from replicate analyses. Instrument calibration was carried out using multi-point calibration curves prepared from certified standard solutions, with linearity verified prior to and during the analytical sequence. Quality control procedures included the analysis of procedural blanks to assess potential contamination, as well as repeated measurements of selected samples to ensure reproducibility. The consistency between results obtained using independent analytical approaches (extraction–photometric method and atomic absorption spectroscopy) was additionally used as an internal validation of data reliability.

The detection limits (LOD) and quantification limits (LOQ) for the analyzed elements were determined as a function of the signal-to-noise ratio of 3 and 10, respectively, resulting in an LOD for the analyzed elements of ~0.05 mg/L. Concentrations which were below the detection limit have been reported with “<0.05”.

No certified reference materials were used in this study and analytical accuracy was verified using multiple point calibration curves created from certified standards, procedural blanks, and replicate measurements. Furthermore, the consistency between results obtained from the independent analytical methods of AAS and extraction–photometric methods was used as an internal validation of the reliable nature of the obtained data.

## 4. Conclusions

This study demonstrates that bottom sediments of anthropogenically impacted freshwater systems exhibit pronounced spatial heterogeneity, with metal concentrations varying by up to 2–3 times between shoreline and central depositional zones. The highest levels of contamination were identified in the Chizhovskoye reservoir, where elevated concentrations of Cu, Zn, Cr, and Ni indicate intensive anthropogenic loading.

It is shown that metal speciation and binding forms, rather than total concentrations, govern environmental behavior and ecological effects. Despite elevated metal contents, no significant phytotoxic effect was observed, confirming that accumulation in plant tissues does not directly correspond to toxicity. This reflects the predominance of relatively stable organo-mineral fractions limiting the availability of biologically active metal species.

The results further indicate that conventional assessments based solely on total metal concentrations may lead to misinterpretation of environmental risk. The observed discrepancies between total content, mobile forms, and biological response highlight the necessity of integrated evaluation approaches combining chemical speciation and bioassays for the reliable assessment of sediment-associated contamination.

## Figures and Tables

**Table 1:** Characteristics of the bottom sediment samples.

Location	Color	The Odor	Consistency	W, %	A, %
r. Loshitsa	black-gray	oily	soft	54.90 ± 2.1	87.62 ± 3.4
Chizhovskoe reservoir	black	oily	soft	65.77 ± 2.8	83.44 ± 2.9
r. Svisloch (zoo)	black	oily	gravelly	21.68 ± 1.3	95.00 ± 2.2
r. Titovka	black	oily	Fluid/silty	74.09 ± 3.1	82.12 ± 3.5
lake Sergeevskoe	black	earthy	Fluid/silty	91.91 ± 4.2	24.24 ± 1.8

**Table 2:** Heavy metal concentrations in water samples, mg/L.

Location	Cu	Zn	Pb	Cr	Ni	Mn
r. Loshitsa	0.15 ± 0.008	0.235 ± 0.002	<0.05	<0.05	<0.05	2.1 ± 0.12
Chizhovskoe reservoir	0.19 ± 0.012	0.190 ± 0.018	<0.05	<0.05	<0.05	2.4 ± 0.14
r. Titovka	0.022 ± 0.010	0.300 ± 0.004	<0.05	<0.05	<0.05	3.2 ± 0.18
lake Sergeevskoe	0.066 ± 0.011	0.018 ± 0.006	<0.05	<0.05	<0.05	0.9 ± 0.07
Note: Values reported as “<0.05” indicate concentrations below the limit of detection						

**Table 3:** Heavy metal concentrations obtained by the combined method, mg/kg.

**Gross Content**						
Location	Cu	Zn	Pb	Cr	Ni	Mn
Loshytsa, riverbank	77	263	19	8	9	418
Loshytsa, riverbed	43	153	14	17	12.5	334
Chizhovskoye, riverbank	157	755	35.8	379	106	425
Chizhovskoye, center	57	462	28	145.5	54.1	392
Titovka, riverbank	13	136	12.9	9	7	480
Titovka, riverbed	23.9	102	12	14	7	359
Sergeevichi, riverbank	14	51	11	4	3	348
Sergeevichi, center	21	74	11	3	2	405
**Mobile Content**						
Location	Cu	Zn	Pb	Cr	Ni	Mn
Loshytsa, riverbank	2	19	–	–	–	104
Loshytsa, riverbed	1.8	13	–	–	–	76
Chizhovskoye, riverbank	2	91.8	–	–	–	108
Chizhovskoye, center	1.4	56.6	–	–	–	102
Titovka, riverbank	0.3	11.6	–	–	–	116.6
Titovka, riverbed	0.3	11.2	–	–	–	106
Sergeevichi, riverbank	6.8	8	2	–	–	104
Sergeevichi, center	8.6	7.4	2.6	–	–	62

**Table 4:** Heavy metal concentrations obtained by the atomic absorption spectroscopy method, mg/kg.

**Gross Content**						
Location	Cu	Zn	Pb	Cr	Ni	Mn
Loshytsa, riverbank	76.8	263	18.6	7.1	8.6	418
Loshytsa, riverbed	43.2	153	14.3	17.6	12.1	334
Chizhovskoye, riverbank	158.3	755	35.9	379	106	425
Chizhovskoye, center	60.1	462	28.7	145.5	54.1	392
Titovka, riverbank	10.7	136	10.9	7.9	5.7	480
Titovka, riverbed	24.2	102	10.1	13.6	6.5	359
Sergeevichi, riverbank	11.2	52	11.3	3.4	3.4	348
Sergeevichi, center	21.9	74.5	10.4	2.5	2.5	405
**Mobile Content**						
Location	Cu	Zn	Pb	Cr	Ni	Mn
Loshytsa, riverbank	2.6	18.2	–	–	–	103.6
Loshytsa, riverbed	1.68	12.6	–	–	–	76.2
Chizhovskoye, riverbank	1.98	91.8	–	–	–	107.8
Chizhovskoye, center	1.3	56.6	–	–	–	101
Titovka, riverbank	0.26	11.2	–	–	–	116.4
Titovka, riverbed	0.28	11	–	–	–	105.4
Sergeevichi, riverbank	6.84	7.8	2	–	–	103.8
Sergeevichi, center	8.56	7.4	2.6	–	–	61.4

**Table 5:** Concentration of heavy metal, associated with organic matter, mg/kg.

Location	Cu	Zn	Pb	Cr	Ni	Mn
Loshytsa, riverbank	53	144	1	2	2	128
Loshytsa, riverbed	23	91	4	6	7.5	130
Chizhovskoye, riverbank	107	435	11	170	46	225
Chizhovskoye, center	30	262	17	90	23	192
Titovka, riverbank	9	106	4	2	1.7	180
Titovka, riverbed	12	92	1	6	2.5	160
Sergeevichi, riverbank	9	45	11	0.9	1	200
Sergeevichi, center	21	64.5	7	0.1	2.5	155

**Table 6:** Mobility of heavy metals between gross and mobility forms, %.

Water Object	Cu	Zn	Pb	Cr	Ni	Mn
river Loshitsa, shore	1.69	3.46	-	-	-	12.39
river Loshitsa, track	1.94	4.12	-	-	-	11.41
Chizhovsky reservoir, shore	0.63	6.08	-	-	-	12.68
Chizhovsky reservoir, center	1.08	6.13	-	-	-	12.88
river Titovka, shore	1.21	4.12	-	-	-	12.13
river Titovka, track	0.58	5.39	-	-	-	14.68
lake Sergeyevskoye, shore	30.54	7.50	8.85	-	-	14.91
lake Sergeyevskoye, center	19.54	4.97	12.50	-	-	7.58

**Table 7:** Values of the heavy metal geoaccumulation index of the bottom sediment samples.

Location	I_geo_ (Cu)	I_geo_ (Zn)	I_geo_ (Pb)	I_geo_ (Cr)	I_geo_ (Ni)	I_geo_ (Mn)
Loshytsa, riverbank	0.37	0.1	0.25	0.55	0.61	0.017
Loshytsa, riverbed	0.16	0.06	0.25	**1.1**	0.96	0.014
Chizhovskoye, riverbank	0.43	0.12	0.3	**1.68**	**1.32**	0.016
Chizhovskoye, center	0.18	0.08	0.31	**1.92**	**1.53**	0.014
Titovka, riverbank	0.2	0.09	0.2	0.58	0.49	0.017
Titovka, riverbed	0.14	0.06	0.21	**1.01**	0.72	0.014

## Data Availability

All data, models, and code generated or used during the study appear in the submitted article.

## Supplementary Materials

The following supporting information can be downloaded at: https://www.mdpi.com/article/10.3390/molecules31081366/s1, Table S1: Main characteristics of the predominant HMs; Table S2: Classification of the geoaccumulation index and pollution level. References [23,24,25,26,27,28] are cited within Supplementary Materials.
