# Supplementary material for: Assessment of Heavy Metal Forms and Mobility in Bottom Sediments of Anthropogenically Impacted Freshwater Bodies in Belarus
Source: Molecules. 2026 Apr 21;31(8):1366. doi: 10.3390/molecules31081366 (PMC13118564; doi:10.3390/molecules31081366)
Supplement: Supplementary file 1 [file molecules-31-01366-s001.zip › molecules-4170433-supplementary.pdf]

# Assessment of heavy metal forms and mobility in bottom sediments of anthropogenically impacted freshwater bodies in Belarus

**Elizaveta Dorozhko [1,*], Witold Kwapinski [2], Valentin Romanovski [3,*]**

[1] Institute of Nature Management of the National Academy of Sciences of Belarus. 220076, Francisk Skorina, 10, Minsk, Belarus

[2] Department of Chemical Sciences, Bernal Institute, University of Limerick, Limerick, Ireland

[2] Department of Materials Science and Engineering, University of Virginia, Charlottesville, VA, 22904, USA

* Corresponding author:

Elizaveta Dorozhko: elizaveta2002belstu@gmail.com

Valentin Romanovski: rvd9ar@virginia.edu

**Table S1.** Main characteristics of the predominant HMs.

| Metal | Form of entry and accumulation | Admission routes | Toxic effects |
|---|---|---|---|
| Cu | $Cu^{2+}$, $(CuHCO_3)^+$ , $Cu(HCO_3)_2$, $CuCl_2$, $CuCl^+$, $CuSO_4$, $(Cu(SO_4)_2)^{2-}$, $(CuO_2)_2$, $CuOH^+$, $Cu(OH)_2$, CuS, $Cu_2O$, $CuCO_3$ | Natural processes involving rocks and soils, wastewater from mining, metallurgical, and chemical enterprises; emissions from non-ferrous metallurgy enterprises (98.7% of all anthropogenic Cu emissions); combustion of leaded gasoline. [23] | For humans, a toxic dose of copper is more than 250 mg: this can lead to liver damage with the development of cirrhosis and secondary brain damage associated with a hereditary disorder of copper and protein metabolism (Wilson-Konovalov disease). |
| Zn | $Zn^{2+}$, $ZnCl_2$, ZnS, $Zn(OH)_2$, $Na_2[Zn(OH)_4]$, ZnO | Zinc enters natural waters as a result of natural processes of weathering and dissolution of rocks and minerals (sphalerite, zincite, goslarite, smithsonite, calamine), as well as with wastewater from ore processing plants and electroplating workshops, parchment paper production, mineral paint production, viscose fiber production, etc. Zinc is used in galvanizing iron, in alloys (brass); in batteries and as a stabilizer for polymers. The natural source of zinc is minerals (sphalerite). [24] | The toxic dose of zinc for humans (with chronic exposure) is 150-600 mg, the lethal dose is 6 g. Immune system disorders, autoimmune reactions; Disorders of the skin, hair, and nails; Painful sensitivity of the stomach, nausea; Decreased levels of iron, copper, and cadmium in the body; |
| Pb | $Pb^{2+}$, $PbCl_2$, PbBrCl, 2PbO- | Lead present in the atmosphere, along with dust, is deposited by precipitation and begins to concentrate in the soil. Plants absorb | The toxic dose of lead for humans is 1 mg, the lethal dose is 10 g. |

| | | | |
|---|---|---|---|
| | $PbBrCl$, $PbSO_4$, $PbS(PO_4)_2$, $Pb(CH_3COO)_2$, $(C_2H_5)_4Pb$ | lead from the soil, natural waters, and atmospheric deposition, while animals ingest it by consuming plants and water. Lead enters the human body through food, water, and dust.<br>The main sources of lead pollution in the biosphere are various engines whose exhaust gases contain tetraethyl lead, thermal power plants that burn coal, and the mining, metallurgical, and chemical industries. A significant amount of lead is also introduced into the soil through wastewater. [25] | Saturnism (lead poisoning): impaired protein synthesis in the blood (anemia), damage to the kidneys, brain (decreased intellectual abilities, aggressive behavior, convulsions) and peripheral nervous system (especially muscle nerves), hearing loss, growth retardation.<br>It has the ability to cross the placenta and accumulate in breast milk.<br>It enhances the toxic effect of other metals. |
| Ni | $Ni^{2+}$, $(NiHCO_3)^+$, $(NiNH_3)^{2+}$, $Ni(H_2O)_2^{6+}$, $(NiNO_3)^+$, $NiOH^+$, $NiCl^+$, $(Ni(SO_4)_2)^{2-}$, $NiSO_4$, $Ni(OH)_2$, $NiCl_2$, $NiSO_4$, $Ni(NO_3)_2$, $Ni(HCO_3)_2$, $NiCO_3$ | The largest source of nickel in the atmosphere is the combustion of diesel fuel and the burning of coal.<br>Other sources of nickel entering the environment as a result of anthropogenic activity, in addition to coal combustion, include metal mining and smelting, the use of diesel fuel and fuel oil, waste incineration, wastewater, Ni-Cd batteries, electroplating, the use of Ni-containing fertilizers, and use in various industries, including as catalysts. [26]<br>Emissions from non-ferrous metal enterprises (97% of all anthropogenic Ni emissions); fuel combustion. | Dermatitis, eczema, vitiligo, respiratory diseases, asthmatic bronchitis, bronchial asthma, astheno-neurotic disorders, impaired protein, DNA and RNA synthesis, impaired cardiovascular function.<br>Nickel compounds belong to group 1 carcinogens: cancer of the oral cavity, throat, lungs, bronchi, kidneys, large and small intestines, sarcoma. |
| Cr | $Cr^{+3}$, $CrO_2^{2-}$, $Cr_2O_7^{2-}$, $Cr_2O_4^{2-}$ | The main anthropogenic sources of chromium entering the environment are cement production plants, glass manufacturing, | Dermatitis, eczema, allergic reactions; irritation of the upper respiratory tract, asthmatic |

| | | | |
|---|---|---|---|
| | $[Cr(H_2O)_6]^{3+}$, $Cr(OH)^{2+}$, $Cr(OH)_2^+[Cr(OH)_4]$ | fuel combustion, ferrous metallurgy, and the metalworking industry.<br>Emissions from ferrous and non-ferrous metallurgy enterprises (alloying additives, alloys, refractories), and mechanical engineering (electroplating) also contribute to chromium pollution. [27] | bronchitis, bronchial asthma, diffuse pneumosclerosis; astheno-neurotic disorders (headache, weakness, dyspepsia, weight loss, etc.); impaired functions of the stomach (gastritis, peptic ulcer), liver (hepatitis), pancreas.<br>Cr(VI) and Cr(III) compounds are carcinogenic: lung cancer, bronchial cancer. |
| Mn | $Mn^{+2}$, $Mn^{+4}$, MnS, $MnSO_4$, $MnCO_3$, $Mn(OH)_2$ | Manganese enters the atmosphere from emissions from ferrous metallurgy, machine building and metalworking, and non-ferrous metallurgy enterprises.<br>Emissions from ferrous metallurgy enterprises (60% of all Mn emissions), machine building and metalworking (23%), non-ferrous metallurgy (9%), and minor sources (welding work, etc.). [28] | Neurotoxic effects (fatigue, drowsiness, decreased reaction time and work capacity, dizziness, depressive and suppressed states), progressive damage to the central nervous system; pneumonia; disruption of calcification processes and the internal structure of bones; toxemia of pregnancy; development of idiocy in embryos. |

**Table S2.** Classification of the geoaccumulation index and pollution level

| $I_{geo}$ | Class $I_{geo}$ | Level of sediment contamination |
|---|---|---|
| < 0 | 0 | Uncontaminated |
| 0–1 | 1 | Between uncontaminated and moderately contaminated |
| 1–2 | 2 | Moderately contaminated |
| 2–3 | 3 | Between moderately and heavily contaminated |
| 3–4 | 4 | Heavily contaminated |
| 4–5 | 5 | Between heavily and extremely contaminated |
| > 5 | 6 | Extremely contaminated |
